# Quantifying Preferences for the Natural World Using Monetary and Nonmonetary Assessments of Value

**DOI:** 10.1111/cobi.12215

**Published:** 2014-01-01

**Authors:** MARTIN DALLIMER, DUGALD TINCH, NICK HANLEY, KATHERINE N IRVINE, JAMES R ROUQUETTE, PHILIP H WARREN, LORRAINE MALTBY, KEVIN J GASTON, PAUL R ARMSWORTH

**Affiliations:** * Department of Food and Resource Economics, and Center for Macroecology, Evolution and Climate, University of CopenhagenRolighedsvej 23, 1958, Copenhagen, Denmark; † Economics Division, University of StirlingStirling, United Kingdom; ‡ Institute of Energy and Sustainable Development, De Montfort UniversityLeicester, United Kingdom; § Department of Animal and Plant Sciences, University of SheffieldSheffield, United Kingdom; ** Environment and Sustainability Institute, University of ExeterCornwall, United Kingdom; †† Department of Ecology and Evolutionary Biology, University of TennesseeKnoxville, TN, U.S.A.

**Keywords:** choice modeling, ecosystem services, psychological well-being, stated preference, urban ecology, valuation

## Abstract

Given that funds for biodiversity conservation are limited, there is a need to understand people’s preferences for its different components. To date, such preferences have largely been measured in monetary terms. However, how people value biodiversity may differ from economic theory, and there is little consensus over whether monetary metrics are always appropriate or the degree to which other methods offer alternative and complementary perspectives on value. We used a choice experiment to compare monetary amounts recreational visitors to urban green spaces were willing to pay for biodiversity enhancement (increases in species richness for birds, plants, and aquatic macroinvertebrates) with self-reported psychological gains in well-being derived from visiting the same sites. Willingness-to-pay (WTP) estimates were significant and positive, and respondents reported high gains in well-being across 3 axes derived from environmental psychology theories (reflection, attachment, continuity with past). The 2 metrics were broadly congruent. Participants with above-median self-reported well-being scores were willing to pay significantly higher amounts for enhancing species richness than those with below-median scores, regardless of taxon. The socio-economic and demographic background of participants played little role in determining either their well-being or the probability of choosing a paying option within the choice experiment. Site-level environmental characteristics were only somewhat related to WTP, but showed strong associations with self-reported well-being. Both approaches are likely to reflect a combination of the environmental properties of a site and unobserved individual preference heterogeneity for the natural world. Our results suggest that either metric will deliver mutually consistent results in an assessment of environmental preferences, although which approach is preferable depends on why one wishes to measure values for the natural world.

## Abstract (ES)

Preferencias de Cuantificación para el Mundo Natural Usando Estudios de Valor Monetario y No Monetario.

**Resumen:**

Dado que los fondos para la conservación de la biodiversidad son limitados, hay una necesidad de entender las preferencias de la gente por sus diferentes componentes. A la fecha, estas preferencias se han medido principalmente en términos monetarios. Sin embargo, cómo la forma en que la gente valora la biodiversidad puede diferir de la teoría económica y hay poco consenso sobre si las métricas monetarias siempre son apropiadas o si el grado al cual otros métodos ofrecen perspectivas alternativas y complementarias sobre el valor. Usamos un experimento de opción para comparar las cantidades monetarias que los visitantes recreativos a las áreas verdes urbanas están dispuestos a pagar para la mejoría de la biodiversidad (incremento en la riqueza de especies de aves, plantas y macroinvertebrados acuáticos) con las ganancias psicológicas auto-reportadas en el bienestar derivado de las visitas a estos mismos sitios. Los estimados de la disponibilidad para pagar (DPP) fueron significativos y positivos; los que respondieron reportaron una ganancia alta en el bienestar a lo largo de tres ejes derivados de las teorías de psicología ambiental (reflexión, apego, continuidad con el pasado). Las dos medidas en general fueron congruentes. Los participantes con bienestar auto-reportado con puntaje por encima de la media estaban dispuestos a pagar cantidades significativamente más altas para mejorar la riqueza de especies, sin importar el taxón, que aquellos participantes con puntaje por debajo de la media. El trasfondo demográfico y socioeconómico de los participantes tuvo una involucración mínima en determinar si el bienestar o la probabilidad de elegir una opción de pago dentro del experimento de opciones. Las características ambientales a nivel de sitio sólo estuvieron un poco relacionadas con la DPP pero demostraron asociaciones fuertes con el bienestar auto-reportado. Ambos acercamientos probablemente reflejen una combinación de las propiedades ambientales de un sitio y la heterogeneidad de la preferencia individual no-observada para el mundo natural. Nuestros resultados sugieren que cualquiera de las dos medidas entregará resultados mutuamente consistentes en un estudio de preferencias ambientales, aunque cuál acercamiento es preferible depende de las razones por las que alguien desea medir los valores del mundo natural.

## Introduction

The natural environment is central to human well-being through its role in ecosystem service provision (Sachs et al. 2009). With limited resources available for its conservation, there is a need to understand people’s preference for different aspects of the natural world as one means to prioritize conservation actions. A commonly used approach is to assign monetary values to changes in ecosystems and the services they supply (e.g., Naidoo et al. 2008; Hanley & Barbier 2009) thereby facilitating making direct comparison with other costs and benefits in decision-making processes (Kahneman & Sugden 2005; Kumar 2010; Whiteman et al. 2010). However, it is increasingly apparent that people’s perceptions of the value of nature may be different from the standard model of economic value. Objections to monetary valuation of nature fall into 3 broad categories: the full value of the natural world cannot be usefully measured in terms of money; if priced in monetary terms, the perceived importance or value of nature is somehow diminished; and it remains unclear what many of the monetary amounts generated by valuation exercises actually mean, either in principle or in practice (Kahneman & Sugden 2005; Aldred 2006; Spash & Vatn 2006; Zendehdel et al. 2008; Spangenberg & Settele 2010). However, recent exercises have demonstrated how attaching monetary values to biodiversity and ecosystem services can provide worthwhile information to decision makers and can help build public and government support for conservation (Kumar 2010; UKNEA 2011).

Nevertheless, a broader range of values may need to be considered (e.g., U.S. Environment Protection Agency 2009) because a central criticism is that attaching a monetary value to biodiversity and the natural world collapses multidimensional values of an object into something with a single cardinal axis (Aldred 2006). For example, at least 6 aspects of cultural ecosystem services have been identified (cultural identity, heritage values, spiritual services, inspiration, aesthetic appreciation, recreation, and tourism) (MEA 2005). Although not all elements can be valued in monetary terms (UKNEA 2011), a full account of the cultural value of the natural world would require that all are at least considered. Further, economic valuation may not be appropriate for all facets of environmental goods, especially with regard to the nonuse values (Nunes & van den Bergh 2001). Indeed, other aspects of services are still more difficult to address, and the money amounts generated through an economic valuation framework may not capture the full value of ecosystems to beneficiaries (e.g., the role of biodiversity in maintaining system resilience) (Walker et al. 2008; García-Llorente et al. 2011). For biodiversity and the natural world, there is still little consensus on when purely monetary metrics are appropriate or on the degree to which other techniques, such as subjective assessments of well-being, will offer markedly different perspectives on value (Liu et al. 2010). Such debates are taking place while policy makers are exploring methods for quantifying personal and societal well-being that fall outside traditional economic measures (CEC 2009; CMEPSP 2009; ONS 2012b).

While there are important conceptual differences between monetary valuation and alternative metrics, the choice of approach often hinges on why values are needed. If the purpose is to inform a benefit–cost analysis of a project or policy which will have impacts on the natural world, then a monetary valuation of environmental changes is needed if they are to be included within the analysis. On the other hand, if we wish to uncover the extent to which a protected area contributes to well-being or to rank alternative management actions in terms of their impact on well-being, then nonmonetary measures will be adequate (although monetary measures could also be used).

A second key distinction between monetary valuation and many nonmonetary measures is that monetary values for the environment are only defined over some change in quantity or quality (whether actual or hypothetical). In contrast, nonmonetary metrics may be attached to the value of the site itself, independent of any actual or potential change.

We compared people’s valuation of the natural world derived from the 2 distinct methodological standpoints. We compared monetary amounts recreational users of urban green spaces were willing to pay for enhancements to species richness with their psychological well-being gains from the current condition of the same sites. We use the stated-preference technique of choice experiments to derive estimates of willingness to pay (WTP) (a standard measure of the economic value of a good to an individual) for enhancements to biodiversity. With this method, people’s WTP is assessed based on discrete choice comparisons that include varying payment levels for defined increases in biodiversity (e.g., Hanley & Barbier 2009; Kumar 2010). In comparison, we used 3 metrics to estimate psychological well-being that recreational visitors reported regarding their emotional attachments and associations with the green spaces. Our choice of well-being metrics has a clear basis in environmental psychology (Proshansky et al. 1983; Kaplan & Kaplan 1989; Altman & Low 1992) and models of holistic health (Engel 1977). They are centered around the premise that the natural world offers people opportunities for reflection, development of positive emotional bonds, and a sense of identity. Based on responses to statements such as “being here makes me feel more connected to nature” and “I feel happy when I am here,” we estimated psychological well-being gains (reflection, attachment, and continuity with the past) derived from people’s interactions with green spaces.

We tested the hypothesis that people have broadly similar preferences regardless of the measure of preference (monetary or psychometric) (i.e., both methods generate a consistent sorting of individuals according to how much they value the natural world). Possible outcomes included neither method elicits positive values for urban green spaces; the 2 methods deliver contrasting results (i.e., people have a significant WTP, but do not report high well-being gains or those people with high well-being gains do not have similarly high WTP), thereby undermining conclusions based on one technique alone; or there is congruence (positive correlation) between the 2 metrics of people’s preferences. Given our design, plausible explanations for the latter option include the socio-economic and demographic characteristics of the respondents were responsible for the observed variation in both data sets (e.g., for WTP; Christie et al. 2006; Jacobsen & Hanley 2009); visitors responded to the natural characteristics of the green space they were visiting (e.g., for well-being; Dallimer et al. 2012); and variation in responses reflected heterogeneous individual preferences for nature conservation. We test for any such associations and examine the extent to which the contrasting approaches may deliver congruent answers.

## Methods

### Study Area

We used Sheffield, a large city in England (human population: 522,700) (ONS 2012a), as our study system. As Sheffield lies at the confluence of several rivers, riparian areas offer an important recreational resource for the city’s residents, especially as they are distributed throughout the urban, suburban, and more rural periphery. Thirty-four sites with public access spanning a wide geographic area were selected to represent the range of riparian green spaces available to city dwellers (Supporting Information).

### Questionnaire Development

We developed a questionnaire to describe the underlying socio-economic and demographic characteristics of the participant and to derive estimates for self-reported psychological well-being gain for individual recreational visitors to each site and WTP for enhancements to the biodiversity (bird, plant, and aquatic macroinvertebrate species richness) at those same sites. These 2 measures of value differed in whether a monetary or nonmonetary metric applied and in whether they valued sites in their current condition or on the basis of changes to the sites. We followed standard practice in such questionnaire designs and placed Likert-scale questions prior to a stated preference valuation exercise (e.g., Bateman et al. 2002). Thus, all respondents were presented with the psychometric statements before the choice experiment. This raises the possibility that responses were influenced by ordering effects (Clark & Friesen 2008) (i.e., positive or negative answers to one set of questions primed respondents to answer in the same way later in the survey). We minimized this potential problem by encouraging respondents to read through and answer both sets of questions by themselves. Interviewers were therefore unaware of how well-being statements had been answered prior to respondents completing the choice experiment. Any potential biases were further limited by ensuring interviewers only provided instruction on how to complete the questionnaire and did not intimate that one set of responses was more appropriate. We wished to engage with as wide a range of people using the green spaces as possible. Each site was therefore visited at least 4 times, covering daytime and early evening, during weekends and weekdays. We used a rule of thumb of approaching every third person. The questionnaire was delivered face-to-face in situ to 1108 visitors (54.3% response rate; median 34 per site) during fall 2009 (Dallimer et al. 2012) by 5 trained interviewers. Interviews took up to 15 minutes to complete and a consistent method of guiding visitors through the questions was used. Prior to starting, each participant was given a brief, scripted, project description (Supporting Information) and an assurance of anonymity. Informed consent was obtained verbally, participation was voluntary, and no compensation was provided. Respondents were predominantly of European ethnicity (91.7%; in line with the population of Sheffield 91.2%), represented both genders well (62% male), and covered a broad age (16 to 70+) and household income range (<£10,000 to >£70,000 per annum).

### Self-Reported Psychological Well-Being Gain

Closed-ended well-being questions were framed around the green space in its current form and were based on the premise that the natural environment may facilitate cognitive restoration and reflection (Kaplan & Kaplan 1989), emotional attachments (Altman & Low 1992), and identity (Proshansky et al. 1983). Seven items measured self-reported reflection; 14 items assessed self-reported emotional attachment and personal identity (see Fuller et al. [2007] and Dallimer et al. [2012] for discussions of the theoretical frameworks and origination of measures). All 21 well-being items were based on a 5-point Likert scale (1 = *strongly disagree*, 3 = *neutral*, 5 = *strongly agree*) in response to the stem question “Please indicate how much you agree with each statement about this stretch of river and the neighbouring banks.” *Stretch of river* referred to the immediate area of river and river banks where the interview was taking place.

We used factor analysis (Tabachnick & Fidell 2001) to identify meaningful subsets of statements that measured a single interpretable factor of well-being. Separate factor analyses were conducted on statements measuring reflection and statements measuring sense of place. The final interpretation of a factor was informed by theory and previous research. We categorized responses (Dallimer et al. 2012) as reflection (opportunity to think and gain perspective); attachment (degree of emotional ties with the stretch of river); or continuity with past (extent to which sense of identity is linked to the stretch of river through continuity across time). Continuous measures were derived by calculating the participant’s average rating of the set of statements forming each factor.

We tested for the effects of 4 socio-economic and demographic variables (age, income, gender, frequency of visits) (Supporting Information) on the respondent’s psychological well-being by including all 4 variables in an analysis of variance (ANOVA) in which the well-being axes were response variables. Post hoc tests were used to determine which categories differed significantly.

### Willingness to Pay

To allow a direct comparison, WTP values were derived for the same sample of participants as the self-reported well-being measures. The methodology adopted was the stated preference nonmarket valuation technique of the discrete choice experiment, which draws upon Lancaster’s (1966) economic theory of value and hedonic price theory (Rosen 1974). The methodology is based on probabilistic choice, where individuals are assumed to choose a single alternative which maximizes their utility from a set of available alternatives. Choice experiments involve presenting participants with a number of choice sets consisting of 2 or more alternatives from which their preferred option is chosen. Each choice is described by various levels of a set of attributes, including a monetary cost which would finance changes in attribute levels and allow the estimation of WTP for changes in the attributes. Choice experiments are commonly used to value changes in riparian systems (Hanley et al. 2006) and biodiversity (Christie et al. 2006). They are consistent with random utility theory and offer a wide range of information on trade-offs among the benefits provided by the different options (Adamowicz et al. 1997, 1998).

Each respondent faced 6 choice sets which asked them to choose between 3 options (Supporting Information). These were 2 policy-on options which included different combinations of the attributes (increases in number of species of birds, plants, and aquatic macroinvertebrates) and a no-cost alternative in which no changes would take place. The policy-on options included the baseline of no change and 2 levels of change (either a 10% or 25% increase) in plant, bird, and aquatic macroinvertebrate richness and 6 levels of cost (£5, £11, £18, £26, £33, £55) specified as increases to the householder’s annual local taxation bill needed to finance the conservation measures. Analyses were conducted in NLOGIT software with a mixed logit specification with an error component model. In all cases, the attributes (increases in species richness across 3 taxonomic groups) included in the experiment were significant and had appropriate signs with positive WTP (Table1).

**Table 1:** Estimated coefficient of willingness to pay (WTP) and mean (SE) WTP in British pounds of recreational visitors to riparian green spaces for enhancements to biodiversity (10% or 25% increase in species richness) for 3 taxonomic groups

			Reflection a		Attachment a		Continuity with past a	
Taxon	Increase (%)	Full model b coefficient, WTP	below median c coefficient, WTP	above median^d^ coefficient, WTP	below median^c^ coefficient, WTP	above median^d^ coefficient, WTP	below median^c^ coefficient, WTP	above median^d^ coefficient, WTP
Birds	10	0.69, 11.99 (0.96)	0.70, 9.58 (1.23)^e^	0.70, 14.87 (1.65)^e^	0.71, 10.09 (1.24)^e^	0.69, 14.21 (1.61)^e^	0.70, 9.78 (1.20)^e^	0.69, 14.51 (1.65)^e^
	25	0.95, 16.51 (0.84)	1.04, 14.31 (1.03)^e^	0.94, 20.02 (1.49)^e^	1.09, 15.38 (1.11)^f^	0.90, 18.61 (1.38)^f^	1.07, 14.93 (1.02)^e^	0.91, 19.14 (1.49)^e^
Plants	10	0.78, 13.48 (0.80)	0.87, 11.93 (0.99)^e^	0.76, 16.25 (1.42)^e^	0.82, 11.61 (0.96)^e^	0.80, 16.40 (1.42)^e^	0.94, 13.15 (0.99)	0.70, 14.68 (1.38)
	25	0.45, 7.86 (0.89)	0.48, 6.59 (1.05)^f^	0.48, 10.15 (1.57)^f^	0.40, 5.69 (1.11)^e^	0.53, 10.96 (1.50)^e^	0.54, 7.52 (1.09)	0.43, 9.02 (1.52)
Aquatic macroinver-	10	0.54, 9.38 (0.92)	0.56, 7.71 (1.14)^e^	0.55, 11.77 (1.59)^e^	0.52, 7.36 (1.19)^e^	0.59, 12.06 (1.53)^e^	0.65, 9.13 (1.32)	0.48, 10.02 (1.58)
tebrates	25	0.69, 11.91 (0.86)	0.77, 10.64 (1.05)^f^	0.66, 14.15 (1.50)^f^	0.73, 10.37 (1.13)^e^	0.70, 14.40 (1.42)^e^	0.74, 10.31 (1.08)^e^	0.69, 14.42 (1.50)^e^
Cost in tax		−0.06 (0.002)	−0.07 (0.003)	−0.05 (0.002)	−0.07 (0.003)	−0.05 (0.002)	−0.07 (0.002)	−0.05 (0.002)
Error component		4.20 (0.19)	3.83 (0.25)	4.46 (0.31)	3.81 (0.26)	4.47 (0.29)	3.66 (0.24)	4.68 (0.33)
Adjusted *R*^2^		0.317	0.321	0.316	0.315	0.320	0.314	0.322
Log likelihood		−4956	−2164	−2484	−2108	−2543	−2169	−2478
Participant sample		1035	484	551	467	568	480	555

a *Self-reported psychological well-being measured on a 1–5 scale (1, strongly disagree; 5, strongly agree)*.

b *All survey participants*.

c *Visitors reporting below median psychological well-being gains*.

d *Visitors reporting above median psychological well-being gains*.

e *Significant differences between WTP estimates for participants reporting above versus below-median well-being at α = 0.05*.

f *Significant differences between WTP estimates for participants reporting above versus below-median well-being at α = 0.1*.

It was not possible to compare directly across coefficients for different subsamples due to scale effects; however, we were able to compare the WTP estimates themselves because the scale parameter canceled out when WTP was calculated. We therefore tested for the effects of the same 4 socio-economic and demographic variables (age, income, gender, frequency of visits) and the environmental attribute of tree cover on the likelihood that a respondent would choose a paying option within the choice experiment. This was done by estimating individual specific parameters which allow the underlying causes of choice heterogeneity to be investigated within the error component model (Supporting Information).

## Results

Respondents expressed a significant positive WTP for enhancements to species richness. For a 10% increase in the number of species, participants were willing to pay £11.99 for birds, £13.48 for plants, and £9.38 for aquatic macroinvertebrates (Table1: full model). For birds and macroinvertebrates, WTP was £16.51 and £11.91, respectively, for a 25% increase in richness. However, for 25% more plant species WTP was reduced to £7.86.

In terms of the psychometric measures, for reflection and attachment over 90% of participants reported well-being of >3 on the Likert response scale. Although the distribution of scores was less skewed for continuity with past, a majority (64%) of respondents recorded well-being gains >3 (Table2). Across all axes, the median well-being was high, ranging from 3.2 for continuity with past to 4.33 for attachment (Table2). Psychological well-being measures were correlated (reflection and continuity with past: *r_s_* = 0.694, *p* < 0.001; reflection and attachment: *r_s_* = 0.699, *p* < 0.001; attachment and continuity with past: *r_s_* = 0.604, *p* < 0.001).

**Table 2:** For 1036 participants who completed the answers to the well-being statements, the median psychological well-being (on a 5-point Likert scale) determined on the basis of participant indications of how much they agreed with each statement about the river and the neighboring banks and the number who reported a mean well-being >3

Variable^a^	Median (lower quartile–upper quartile)	Participant with well-being > 3
Reflection	4.00 (3.57−4.43)	959
Attachment	4.33 (3.83−4.83)	997
Continuity with past	3.20 (2.60−3.80)	665

a *Defined in Table1*.

**Figure 1:**
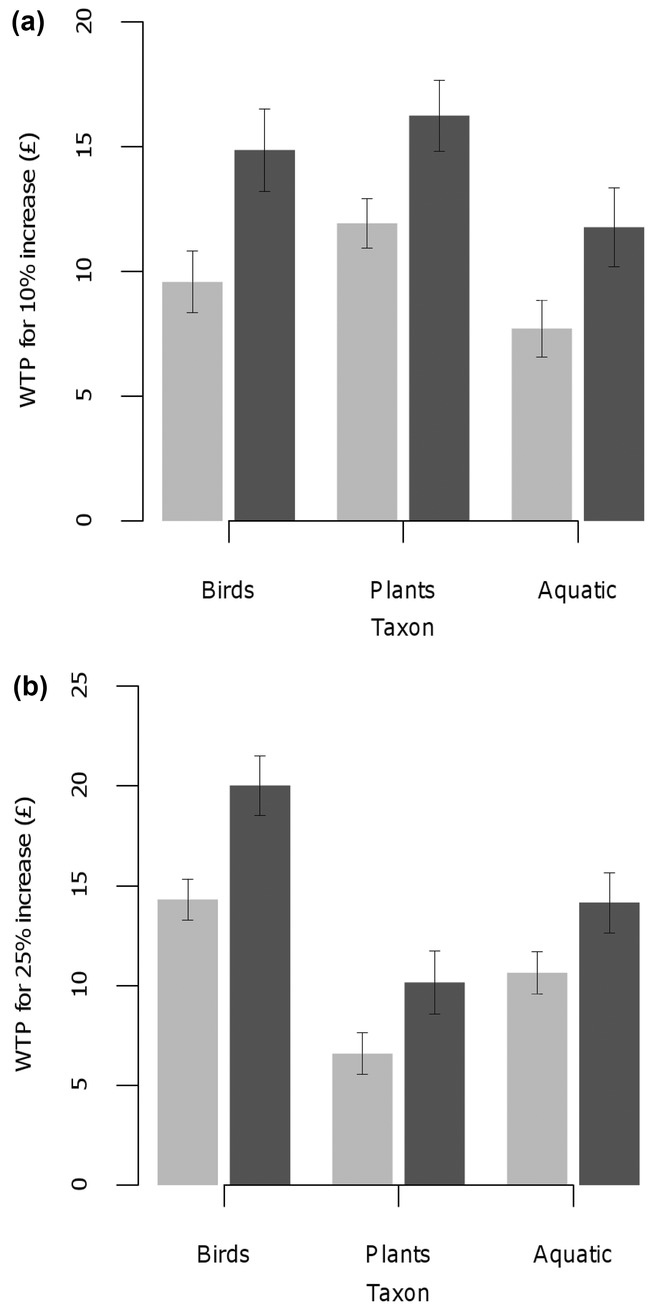
Estimates of mean (error bars are standard errors) willingness to pay (WTP) of recreational visitors to riparian green spaces in Sheffield (U.K.) for (a) 10% and (b) 25% increase in species richness for 3 taxonomic groups (birds, plants, aquatic macroinvertebrates) (dark gray, visitor reporting above-median well-being for the reflection axis; light gray, visitors reporting below-median psychological well-being gains for the reflection axis).

Individuals reporting higher well-being were willing to pay more than those with lower scores (Fig.1 & Table1). For example, participants with above-median reflection scores were willing to pay £20.02 (SE 1.49) for a 25% increase in bird species richness, which is significantly higher (*t* = 3.15, df = 1033, *p* < 0.01) than the £14.31 (SE 1.03) estimated for those with below-median reflection scores. Similarly, participants with high attachment to the green space were willing to pay significantly more for 10% increases in plant (£16.40) and aquatic macroinvertebrate richness (£12.06) than their counterparts who expressed below-median well-being (£11.61 and £7.36; *t* = 2.79 and 2.42 *p* < 0.01 and *p* < 0.05 respectively, df = 1033). Across all well-being axes and taxa, WTP was higher for participants reporting above-median psychological well-being gains from their green space visit (Table1). This demonstrates a positive correlation between the economic and environmental psychological values of the natural world.

There was substantial variation in WTP and well-being estimates across respondents. Nevertheless, there was broad agreement between the 2 metrics, which indicates that a similar set of factors may underlie them. Possible explanations include that across-individual differences in value may primarily reflect individual socioeconomic status (e.g., someone with more income attaches higher well-being to the urban green space); between survey site differences in the environmental characteristics people care about (e.g., someone surveyed in site A has both higher WTP and higher well-being scores than someone surveyed in site B); or factors we did not observe (or even perhaps that are unobservable), such as the heterogeneous preferences respondents have for nature conservation, something that both metrics have been used to assess (e.g., Christie et al. 2006; Dallimer et al. 2012). Having detected meaningful variation in WTP and well-being measures, we test the first 2 of these possibilities.

**Figure 2:**
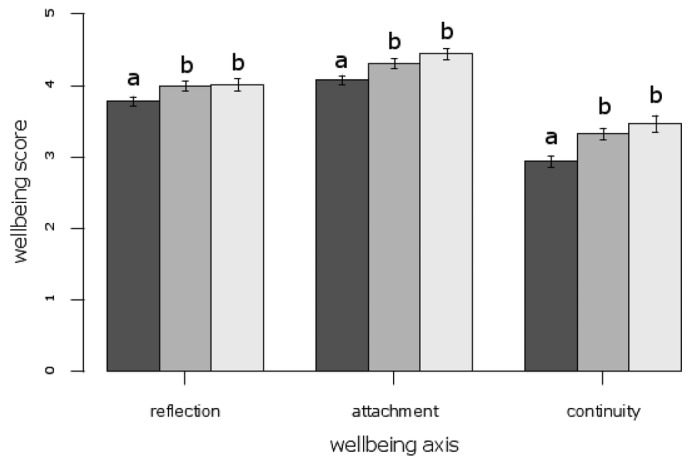
Self-reported psychological well-being of visitors to urban green spaces, measured on 3 axes (reflection, attachment, and continuity with past) and by age category (dark gray, <40 years old; medium gray, 40–60 years old; light gray, >60 years old; error bars, 95% CI; differences between categories are significant if letters are not the same [Supporting Information]).

For the choice experiment, the estimated coefficients derived from an error component model for the socio-economic variables showed that only age significantly influenced choices (Supporting Information). People in the youngest age category (up to 40 years old) were more likely to be willing to pay to enhance species richness. Gender, income, and frequency of visit to the site where the respondent was surveyed did not determine choice to a statistically significant degree. Similarly, across all 3 well-being axes only the effect of age was significant. In contrast, however, those in the youngest age category reported significantly lower well-being than other participants (Fig.2 & Supporting Information). Because respondents self-select in terms of which sites they visit (and thus were sampled at) the relationships between stated choice and well-being and socio-economic variables are conditioned by the self- selection process.

To test whether WTP varied according to the environmental characteristics of sites, we used tree cover as an example because it is an immediately visible element of the natural world. All 3 well-being axes showed significant variation across sites (ANOVA: reflection *F*_1,32_ = 6.097, *p* < 0.05; attachment *F*_1,32_ = 11.92, *p* < 0.01, continuity with past *F*_1,32_ = 8.267, *p* < 0.01) and were positively associated with tree cover (reflection *r* = 0.400, *p* < 0.05, attachment *r* = 0.521, *p* < 0.01, continuity with past *r* = 0.453, *p* < 0.05); there was no evidence of nonlinearity (Supporting Information). Visitors to locations with above-median tree cover were willing to pay a significantly greater amount for a 25% increase in avian species richness (Table3), but were not willing to pay more for other taxa.

**Table 3:** Recreational visitors to riparian green spaces in Sheffield (U.K.), with below and above median tree cover, willingness to pay (WTP) (in British pounds) for enhancements to biodiversity (10% or 25% increase in species richness) for 3 taxonomic groups

Taxon	Increase	Below median^a^ coefficient, WTP (SE)	Above median^b^ coefficient, WTP (SE)
Birds	10	0.75 (0.09), 10.66 (1.27)^c^	0.66 (0.07), 14.32 (1.71)^d^
	25	1.03 (0.75), 14.72 (1.09)^b^	0.92 (0.06), 19.89 (1.62)^b^
Plants	10	0.89 (0.07), 12.69 (0.96)	0.70 (0.07), 15.22 (1.57)
	25	0.57 (0.07), 8.09 (1.05)	0.37 (0.08), 8.04 (1.68)
Aquatic macroinvertebrates	10	0.61 (0.08), 8.70 (1.15)	0.49 (0.07), 10.65 (1.66)
	25	0.84 (0.07), 12.04 (1.10)	0.56 (0.07), 12.03 (1.57)
Cost in tax		−0.070 (0.003)	−0.046 (0.002)
Error component		2.525	2.510
Adjusted *R*^2^		0.249	0.215
Log likelihood		−2121.66	−2321.17
Participant sample		423	442

a *Values are estimated coefficient and mean WTP*.

b *Significant differences between WTP estimates for participants visiting sites with above and below median tree cover, at α = 0.05*.

c *Significant differences between WTP estimates for participants visiting sites with above and below median tree cover, at α = 0.1*.

By carrying out in situ surveys without any explicit comparisons with alternative sites, we potentially limited our ability to separate the importance of respondent characteristics, such as age or income which may affect which site respondents chose to visit, from site characteristics, such as tree cover. However, respondent characteristics had little effect, so it is likely this issue is not a major concern. Nevertheless, as there is the possibility that site visitors are a self-selected subset of the population partly based on their opinions and feelings about the natural world, our sample of respondents is not representative of the wider population and hence our results only apply to recreational visitors to urban green spaces.

## Discussion

Visitors to urban green spaces were willing to pay a significant amount for biodiversity enhancements and reported psychological well-being gains from their visit. Hence, both metrics returned strongly positive values for people’s preferences for the natural world. This was apparent even in urban green spaces, which are likely to be relatively species poor and structurally simple compared to locations (either nationally or globally) that are noted for their conservation interest or scenic beauty. By gathering WTP and self-reported well-being measures from the same individuals at the same time and place, we were able to make a direct comparison between the 2 radically different measures of value. It is conceivable that a negative correlation could exist between the 2 metrics, if for example respondents are willing to pay less for improvements at sites which they value highly, in psychometric terms, in current conditions. However, we found strong evidence of a positive correlation: across 3 well-being measures, 3 taxonomic groups, and 2 levels of species richness increase, participants who reported above median well-being gains for existing sites also expressed a higher WTP for enhancements to biodiversity.

In general, estimates of WTP for biodiversity conservation are positive. Martin-Lopez et al. (2008) compiled mean WTP estimates for species conservation from 60 studies and reported values between US$2.87 and US$206.93. In the United States, valuations for single species varied from $5 to $126 per household per year, and for multiple species ranged from $18 to $194 (Nunes & van den Bergh 2001). This variation is generally believed to be driven by a combination of the socio-economic and demographic characteristics of participants (e.g., Christie et al. 2006; Jacobsen & Hanley 2009). However, we found only limited evidence that the socio-economic and demographic background of participants influenced the likelihood that they would opt to contribute to enhancements to biodiversity. Income, gender, and frequency of visit played no role. Only participants in the lower age category (under 40) were significantly more likely to choose a paying option relative to middle aged respondents within the choice experiment.

Aspects of the type of biodiversity under study also influence people’s WTP for conservation. We anticipated that WTP would be highest for birds because in our study area people are most familiar with this taxon (Dallimer et al. 2012). Although this was the case with WTP for a 25% increase in richness, at the 10% level, WTP was highest for plants. Funds contributed toward a 25% increase in plant richness were, however, lower than for a 10% increase. This perhaps indicates a threshold where perceptions of an overgrown environment in an urban context begin to impact preferences for higher numbers of plant species. Natural landscapes are, in general, preferred to built ones (Kaplan & Kaplan 1989; Herzog et al. 2000). However, preferences for natural elements do not universally extend to urban landscapes (Özgüner & Kendle 2006 and references therein). Confounding factors include characteristics that may indicate a lack of maintenance (Özgüner & Kendle 2006; Kenwick et al. 2009). It is therefore conceivable that people associated a 25% increase in plant species with overgrown or unmaintained habitat. Finally, despite the fact that invertebrates are often unknown to both the general public and policy makers (Cardoso et al. 2011), recreational visitors to riparian green spaces were willing to pay £9.38 and £11.91 for a 10% and 25% increase, respectively, in the number of aquatic macroinvertebrate species present at a site. These significantly positive values were recorded even though the taxon in question is largely unobservable to casual visitors, possibly because they regard macroinvertebrates as indicators of the general ecological health of the rivers.

Self-reported psychological well-being across all 3 axes (reflection, attachment, and continuity with past) was generally high, but remained below the maximum, indicating that the existing condition of sites could be improved. Nevertheless, the large majority of participants who responded positively to the well-being statements was in line with our expectations, given the growing literature documenting the many personal and societal benefits that exposure to the natural environment can provide (Kuo 2001; Fuller et al. 2007; Berman et al. 2008; Bowler et al. 2010; Dallimer et al. 2012; Ward-Thompson et al. 2012). We may expect that respondents would report higher well-being based purely on their socio-economic and demographic background (cf. Blanchflower & Oswald 2004). Broadly speaking, this was not the case here with only participants over the age of 40 reporting significantly higher well-being gains (Fig.2). There was no effect of income, gender or frequency of visit. In contrast, all 3 well-being axes were positively associated with the proportion of tree cover at a site, indicating that, as has previously been demonstrated (Dallimer et al. 2012), site-level characteristics can be important determinants of well-being gains from green spaces.

Eliciting monetary values for elements of the natural world is being increasingly undertaken. For this to be legitimate it must reflect the multiple facets of value that people attach to nature and be in accordance with other recognized measures of value. To determine differences among individuals in the values they place on the natural world, we compared a psychometric measure of current site quality with an economic measure of improved site quality. We found that if respondents felt a strong association with a site they were willing to pay more for its improvement. Perhaps surprisingly, these monetary and nonmonetary measures produced results that were broadly congruent. This result is evident despite the fact that the 2 metrics were not used to measure precisely the same thing. The psychological well-being statements were framed around the site in its current form, while the choice experiment focused on enhancements to species richness. The well-being statements did not address species richness directly; rather, they concentrated on the current condition of the green space more generally.

With the exception of age, socio-economic and demographic variables did not underpin variation in either WTP or psychometric measures of well-being. Variation in WTP and well-being could be largely due to the properties of the environments that the respondents were experiencing. We found some evidence to support this hypothesis. Well-being was positively associated with site-level tree cover, and WTP estimates for a 25% increase in the number of bird species were significantly higher in sites with above-median tree cover. We therefore conclude that variation largely reflected the environmental properties of a site, unobserved preference heterogeneity between individuals, and other unmeasured factors in the experiment. This finding reinforces the validity of both methods and lessens the relevance of debates over which valuation metric is most appropriate to conservation. However, if the goal of a valuation exercise is to contribute to a cost–benefit analysis (e.g., Hanley & Barbier 2009), then only the results of the choice experiment would be appropriate. Nevertheless, if the aim is to assess relative preferences and thus sort people consistently according to how much they value the environment, then as far as we have so far been able to parse out differences, there is little to choose between the metrics. Each offers an alternative, but complementary approach. Indeed, because psychometric well-being is one component of people’s utility function and WTP is derived from this same function, it is not surprising that the 2 metrics send the same signal about the value of the natural environment.

Environmental features, such as those which might distinguish among urban green spaces, are likely to be meaningful when considering how best to manage ecosystem services and biodiversity. Future work could focus on more effectively determining the relative importance of environmental properties of a site and unobserved preference heterogeneity in underpinning people’s preferences and values. Further, we have not addressed whether the psychometric and economic approaches would be equally congruent if we wished to make different relative comparisons, such as preferences among policy options, between sites, or even in deciding which elements of the biota should be prioritized in any conservation initiatives.

Ascribing values to ecosystem services is one way of ensuring that they are given more consideration in decision making and policy development. However, the value of the natural world is multi-dimensional. It therefore makes sense to characterize it more fully and in ways that will be meaningful to many different audiences and stakeholders. As policy makers begin exploring nonfinancial methods for quantifying personal and society well-being that fall outside standard economic measures, there is a need understand the empirical relationships between monetary and nonmonetary measures of environmental quality. Indeed, there is a timely opportunity for the conservation community to emphasize the credibility of the ecosystem service approach through the integration of monetary and nonmonetary metrics for quantifying the value of the natural world.

## Supporting Information

Location of the sampling sites (Appendix S1), detailed methodological description of the choice experiment (Appendix S2), an example choice card (Appendix S3), figures illustrating the association between mean site-level well-being and tree cover (Appendix S4), factor loadings used for the derivation of the well-being axes (Appendix S5), socio-economic and demographic characteristics of respondents (Appendix S6), estimated coefficients from socio-economic variables included in the error component model (Appendix S7), and ANOVAs used to detect differences between the self-reported psychological well-being across participants with different socio-economic variables (Appendix S8) are available online. The authors are solely responsible for the content and functionality of these materials. Queries (other than absence of the material) should be directed to the corresponding author.
